# The Effect of Textbook Analysis as a Teacher Professional Development Tool on Teacher Understanding of Nature of Science

**DOI:** 10.1007/s11191-023-00442-7

**Published:** 2023-05-11

**Authors:** Tarisai Chanetsa, Umesh Ramnarain

**Affiliations:** University of Johannesburg, Johannesburg, South Africa

## Abstract

This article reports on the effect of textbook analysis as a tool of teacher professional development on nature of science (NOS) understanding of 10 science teachers in South Africa. The teacher professional development program (TPDP) was based on an explicit reflective methodology of textbook analysis and conducted online due to the Covid-induced lockdown. NOS understanding of the participant teachers was documented pre-training and post-training using a questionnaire designed by the researchers, termed the IFVNOS questionnaire. This tool was formulated based on the views of nature of science questionnaire version C (VNOSC) and the reconceptualised family resemblance approach (RFN) questionnaire. The same tool was used pre- and post-training. A comparison was made of the pre- and post-training results and it was found that there was a general individual increase in NOS understanding in 9 of the 10 teachers. The creative, scientific knowledge, science methods and ethical practices NOS aspects showed the greatest improvement in understanding by the teachers as a collective, whilst inferential NOS showed no overall change in understanding. This study showed that textbook analysis can be used as a professional development tool to improve NOS understanding of in-service science teachers.

## Introduction

Nature of science (NOS) is a construct that is naively understood by science teachers at all levels of schooling, worldwide and in South Africa (Govender & Zulu, 2017; Gwebu, 2015; Kurup, 2010). This naïve understanding could possibly be attributed to the complexity of defining NOS which researchers have dynamically formulated over decades of years from the 1960s to present date. An understanding of NOS as documented in literature is crucial for the development of scientific literacy, among other benefits (Khishfe, 2022). A scientifically literate citizen has the ability to interact with material of a scientific nature and takes responsible personal decisions based on this material. This study focuses on textbook analysis as a professional development tool aimed at enhancing NOS understanding of science teachers in South Africa. At the time of conducting this study, there were limited opportunities (if any at all) of engaging with NOS in formal settings such as professional development programs.

NOS is a dynamic construct, defined over decades of years by various scholars. There has been much debate about its meaning and what could be an appropriate NOS pedagogy (Brock & Park, 2022). One definition by Lederman et al. (2013) states that NOS is the “epistemology of science, science as a way of knowing, or the values and beliefs inherent to the development of scientific knowledge” (Lederman, 2007, p. 140) in what has become known as the consensus view of NOS. In the 2010s, the consensus view of NOS was challenged by some researchers (e.g. Allchin, 2011; Erduran & Dagher, 2014) for its shortcoming of not taking a holistic approach to defining NOS. Erduran & Dagher (2014) defined NOS as a cognitive-epistemic system within a social-institutional system, in what is known as the reconceptualised family resemblance approach to NOS. NOS is not defined by a single construct but rather it is an assortment of concepts.

Teachers do not generally hold a clear understanding of what NOS really is despite its importance in science education. Researchers have shown that an understanding of NOS translates to the development of scientific literacy (Chaiyabang & Thathong, 2014) and promotes an understanding of the differences between science and other disciplines such as religion, proto-science or indigenous knowledge (Bell, 2008; Dickhaus, 1999). An inclusion of NOS in the classroom promotes interest of learners in science through scientific investigations (Vhurumuku, 2010) and arouses their curiosity (Gwebu, 2015). NOS has formed the basis of curriculum documents worldwide; for example, in Chile (Pavez et al., 2016) in addressing scientific literacy, the NOS aspects that should be taught to learners is explicitly stated. In the USA (Pleasants, 2017), understanding NOS is one of the goals of science education. NOS is also featured in South African school science curriculum documents with the specific aim number 3 of the Physical Sciences curriculum explicitly stating the need for learners to display “an understanding of the nature of science and its relationships to technology, society and environment” (Department of Basic Education (DBE), 2011, p. 8). Understanding NOS is important in science education as stipulated in curriculum documents, but as research will show, it is a naively understood construct. In South Africa, there are little known opportunities for in-service teachers to interact with and expand on NOS ideas (Govender & Zulu, 2017).

Literature articulates that professional development programs aimed at improving NOS understanding of teachers should follow an explicit, reflective methodology (García-Carmona, 2022; McDonald, 2010; Southerland et al., 2006). Explicit approaches to teaching NOS clearly state NOS understanding as an objective, and contexts are provided which allow interaction with the NOS aspects. Argumentation is an example of an explicit reflective methodology in which characteristics of socio-scientific processes, such as global warming, can be mirrored to NOS aspects (Kutluca & Aydin, 2017). Engaging teachers in discussions of socio-scientific issues where they are required to formulate a position based on an issue and defend that position is an explicit approach to teaching NOS. The history of science (HOS) could be used to promote the learning of science content as well as the nature of science (Clough, 2017). The research question addressed is: How does a professional development program on textbook analysis for NOS enhance NOS understanding of science teachers?

## Conceptual Framework

The framework developed for this study is the integrated family resemblance approach (IFVNOS). IFVNOS is based on the consensus view (CV) tenets by Abd-El-Khalick (2012) and the reconceptualised family resemblance approach by Dagher & Erduran (2016). According to Erduran et al. (2019), the aim of FRA, on which RFN is based, is not to teach the individual NOS aspects but rather to present NOS holistically according to a given context. Considering that this study aimed to teach and make changes to the understanding of NOS by in-service teachers, it became apparent that a framework encapsulating the holistic approach to NOS of RFN and explicit tenets of the consensus view needed to be developed.

In developing this framework, the researchers analysed the widely recognised consensus view of NOS which comprises single word or short statements depicting science and added to these tenets categories from RFN which were not represented in the CV. In this way, within RFN categories, tenets of the consensus view on NOS were extracted. A comparison of the categories of RFN and the tenets of NOS conducted by the researchers is documented in the form of a table (see Table 1) to illustrate the understanding of the author on how the consensus view is embedded in RFN.

**Table 1:** CV in RFN

RFN Category	Description	CV NOS tenets embedded
Aims and values	The key cognitive and epistemic objectives of science such as accuracy and objectivity Social, cultural and political values such as honesty, applicability to human needs	• Social and cultural embeddedness of science • Social dimension of science • Creativity • Theory-driven
Methods	Manipulative and non-manipulative techniques underpinning scientific investigations	• Myth of the scientific method
Scientific practices	The set of epistemic and cognitive practices that lead to scientific knowledge through social certification	• Empirical • Inferential • Social dimension of science
Scientific knowledge	Theories, laws, models and explanations that underpin the outcomes of scientific inquiry	• Scientific laws • Scientific theories • Social dimension of science • Tentative
Social certification and dissemination	The social mechanisms through which scientists review, evaluate and validate scientific knowledge through peer review systems of journals	• Social dimension of science
Scientific ethos	The norms that scientists employ in their work as well as in interaction with colleagues	• None
Social values	Values such as freedom, respect for the environment and social utility	• None
Professional activities	How scientists engage in professional settings such as attending conferences and doing public reviews	• Social dimension of science
Social organisations and interactions	How science is arranged in institutional settings such as universities and research institutes	• Social and cultural embeddedness of science
Financial systems	The underlying financial dimensions of science including the funding mechanisms	• Social and cultural embeddedness of science
Political power structures	The dynamics of power that exist between scientists and within science cultures	• Social and cultural embeddedness of science

Two categories of RFN have no consensus view tenet representation, that is, in the ‘scientific ethos’ and ‘social values’ categories. Scientific ethos is defined as the norms that scientists employ in their work as well as in interaction with colleagues, and social values are defined as values that include freedom, respect for the environment and social utility. From the two categories of ‘scientific ethos’ and ‘scientific values’ emerged the keywords “ethical practices” derived from the definitions provided by the two categories. Ethical practices as a keyword were thus included in the conceptual framework. The category ‘scientific knowledge’ from RFN encompassed very well statements on the tenets of ‘theories, laws, models and explanations that underpin the outcomes of scientific inquiry’ from CV, and so was retained from RFN. Similarly, ‘methods’ matched with ‘Myth of the scientific method’ and was retained as an RFN category because it is more encompassing than corresponding CV tenets. Through key word analysis of RFN categories, the researchers formulated short statements mirroring tenets of the CV and added these to the tenets of NOS. The table below lists the 11 IFVNOS aspects constructed by the researchers that form the conceptual framework of the study (Table 2).

**Table 2:** Integrated aspects of NOS (IFVNOS)

NOS aspect derived from consensus view	Definition
Empirical	• Scientific claims are derived from, and/or consistent with, observations of natural phenomena • Scientists, however, do not have “direct” access to most natural phenomena: Their observations are almost always filtered through the human perceptual apparatus, mediated by the assumptions underlying the functioning of “scientific” instruments, and/or interpreted from within elaborate theoretical frameworks
Inferential	There is a crucial distinction between observations and inferences: • Observations are descriptive statements about natural phenomena that are accessible to the senses (or extensions of the senses) and about which observers can reach consensus with relative ease (e.g. objects released above ground level tend to fall to the ground) • Inferences, on the other hand, are statements about phenomena that are not directly accessible to the senses (e.g. objects tend to fall to the ground because of “gravity”) • Scientific constructs, such as gravity, are inferential in the sense that they can only be accessed and/or measured through their manifestations or effects
Creativity	• Science is not an entirely rational or systematic activity. Generating scientific knowledge involves human creativity in the sense of scientists inventing explanations and theoretical entities • The creative NOS, coupled with its inferential nature, entails that scientific entities (atoms, force fields, species, etc.) are functional theoretical models rather than faithful copies of “reality”
Tentative	• Scientific knowledge is reliable and durable, but never absolute or certain. All categories of knowledge (“facts”, theories, laws, etc.) are subject to change. Scientific claims change as new evidence, made possible through conceptual and technological advances, is brought to bear; as extant evidence is reinterpreted in light of new or revised theoretical ideas; or due to changes in the cultural and social spheres or shifts in the directions of established research programmes
Theory-driven	• Scientists’ theoretical and disciplinary commitments, beliefs, prior knowledge, training and expectations influence their work. These factors affect scientists’ choice of problems and methods of investigation, observations (in terms of what is and is not observed), and interpretation of these observations. This (sometimes collective) individuality or mindset accounts for the role of theory in generating scientific knowledge. Contrary to common belief, science never starts with neutral observations. Like investigations, observations are always motivated and guided by, and acquire meaning in light of questions and problems derived from, certain theoretical perspectives
Social dimension of science	• Scientific knowledge is socially negotiated. This should not be confused with relativistic notions of science • This dimension specifically refers to the constitutive values associated with established venues for communication and criticism within the scientific enterprise, which serve to enhance the objectivity of collectively scrutinised scientific knowledge by decreasing the impact of individual scientists’ idiosyncrasies and subjectivities • The double-blind peer-review process used by scientific journals is one aspect of the enactment of the NOS dimensions under this aspect
Social and cultural embeddedness of science	• Science is a human enterprise embedded and practised in the context of a larger cultural milieu. Thus, science affects and is affected by various cultural elements and spheres, including social fabric, worldview, power structures, philosophy, religion and political and economic factors. Such effects are manifested, among other things, through public funding for scientific research and, in some cases, in the very nature of “acceptable” explanations of natural phenomena (e.g. differing stories of hominid evolution have resulted from the advent of feminist perspectives brought about by increased access, participation and leadership of females in the biosocial sciences)
Science vs pseudoscience	• Statements trying to distinguish science from other disciplines of inquiry (e.g. religion, philosophy)
NOS aspect derived from RFN	Definition
Methods	• Manipulative and non-manipulative techniques underpinning scientific investigations
Scientific knowledge	• Theories, laws, models and explanations that underpin the outcomes of scientific inquiry. There is a difference between theories and laws. Theories and laws are different kinds of knowledge, and one does not become the other
Ethical practices	• The norms that scientists employ in their work as well as in interaction with colleagues and values such as freedom, respect for the environment and social utility respectively

## Measuring IFVNOS

To collect views on NOS understanding, the researchers developed the integrated family views of nature of science questionnaire. The questionnaire comprises 12 open-ended questions adopted from the views of NOS (VNOS(C)) by Lederman et al. (2002) and RFN questionnaire by Kaya et al. (2019). Questions on understanding of each of the 11 IFVNOS aspects are represented in the questionnaire as shown in the Table 3 below. The table shows the origin of each question, VNOS(C) or RFN, and the corresponding IFVNOS aspect assessed.

**Table 3:** Sources of IFVNOS questions

IFVNOS	Source	Integrated NOS aspect
1. What, in your view, is science?	VNOS(C)	Science vs. pseudoscience
2. Do all scientific disciplines such as Physics, astronomy, Biology and Chemistry use the same scientific method?	RFN questionnaire	Scientific methods
3. Does the development of scientific knowledge require experiments?	VNOS(C)	Empirical
4. After scientists have developed a scientific theory does the theory ever change?	VNOS(C)	Tentative
5. Describe the purpose of theories, laws and models in producing scientific knowledge	RFN questionnaire	• Scientific theories • Scientific laws
6. How certain are scientists about the structure of the atom? What specific evidence do you think scientists used to determine what an atom looks like?	VNOS(C)	• Creative • Empirical • Theory laden • Inferential
7. Do scientists use their creativity and imagination during their investigations?	VNOS(C)	• Creative • Theory laden
8. How are different conclusions possible if scientists in both groups have access to and use the same set of data to derive their conclusions?	VNOS(C)	• Inferential • Theory laden • Social and cultural embeddedness of science
9. Do you believe that science is universal or does it reflect social and cultural values?	VNOS(C)	• Social and cultural embeddedness of science • Ethical practices
10. Why do scientists engage in professional activities such as attending conferences and doing publications?	RFN questionnaire	Social dimension of science
11. Scientists work in organisations or establishments such as universities and research centres, how are they organised in these institutions?	RFN questionnaire	Social dimension of science
12. Teaching epistemic, cognitive, social and cultural values should be core components of the science curriculum. Do you agree or disagree with this statement?	RFN	• Ethical practices

In the analysis of responses to questions, a second coder was used to ascertain the reliability of the NOS codes assigned by the researcher on all responses from the IFVNOS. The coder selected was an expert in science education, holding a masters’ degree and enrolled as a senior PhD student at the time of writing this thesis. The coder coded and rated a third of the data independently to the researcher. Inter-rater reliability was achieved by comparing and contrasting the treatment of NOS aspects. Initially, 89% agreement was reached between the coders. Any differences were resolved through extended discussions by further reference to the materials until a consensus was reached. The items in the IFVNOS questionnaire are valid due to its origins from the VNOS(C) and RFN instrument that has been validated and verified through numerous revisions. To confirm validity, the IFVNOS was distributed to three researchers in science education. In a pilot study, three teachers were interviewed on their readability of the questions. Now issues regarding readability were revealed from these interviews.

## Method

This is a qualitative case study. Ten science teachers were purposefully selected to participate in the study. Selection was based on willingness to participate, access to Internet online services and their teaching of any science subject at the high school level. In South Africa, all learners take Natural Sciences in the first two years of high school grades 8–9. From grades 10–12, learners opt for Life Sciences and/or Physical Sciences. Five Life Sciences teachers and five Physical Sciences teachers participated in the study. Table 4 below shows the profile of teachers. All teachers have at least a degree qualification to teach science. These include the Bachelor of Education (BEd), Bachelor of Education Honours (BEd (Hons), Bachelor of Science (BSc), Master of Education (MEd), Post-graduate diploma in Education (PGCE) and Higher Diploma in Education (HDE). Seven teachers are female and 3 are male. Their teaching experience ranges from 1.5 to 33 years.

**Table 4:** Profile of teachers

Name	Age	Gender	Qualifications	Teaching experience (years)	Responsibilities
Teacher 1	34	F	BEd (FET), Natural Sciences	13	Curriculum developer GET (NS)
Teacher 2	38	M	BSc (Bio & Stats) BSc Hons (Bio)	11	Life Sciences teacher up to matric and A level Cambridge curriculum
Teacher 3	25	F	BEd(Hons curriculum); MEd	1 year 6 months	Teaching Grade 8 Natural Sciences and Life Sciences up to matric
Teacher 4	57	F	BSc (Applied Chem) HDE	13	Grades 9, 10–12 Physical Sciences teacher Head of Department Science
Teacher 5	48	F	BEd (Bio) MSc (Curriculum Studies and Development)	22	Life Sciences teacher grades 10–12
Teacher 6	50	M	BEd (Bio) Diploma in Education Sciences	25	Grades 10–12 Life Sciences Head of Department
Teacher 7	41	F	BSc, MSc, PGCE	2	Grades 9 and 10 Physical Sciences
Teacher 8	44	F	Dip in Education BCom BEd (Hons) Certificate in Physical Sciences and Mathematical Literacy	22	Grades 10 and 11 Physical Sciences teacher Grades 11 and 12 Mathematical Literacy teacher
Teacher 9	37	F	BEd (FET), Natural Sciences	14	Grades 10–12 Life Sciences
Teacher 10	41	M	BSc, PGCE	15	Grades 10–12 Physical Sciences

BSc is Bachelor of Science; BEd (Hons) is Bachelor of Education Honours, BCom is Bachelor of Commerce; Dip is diploma; PGCE is Postgraduate Certificate in Education; GET is General Education and Training; FET is Further Education and Training; MSc is Masters of Science

**Fig. 1:**
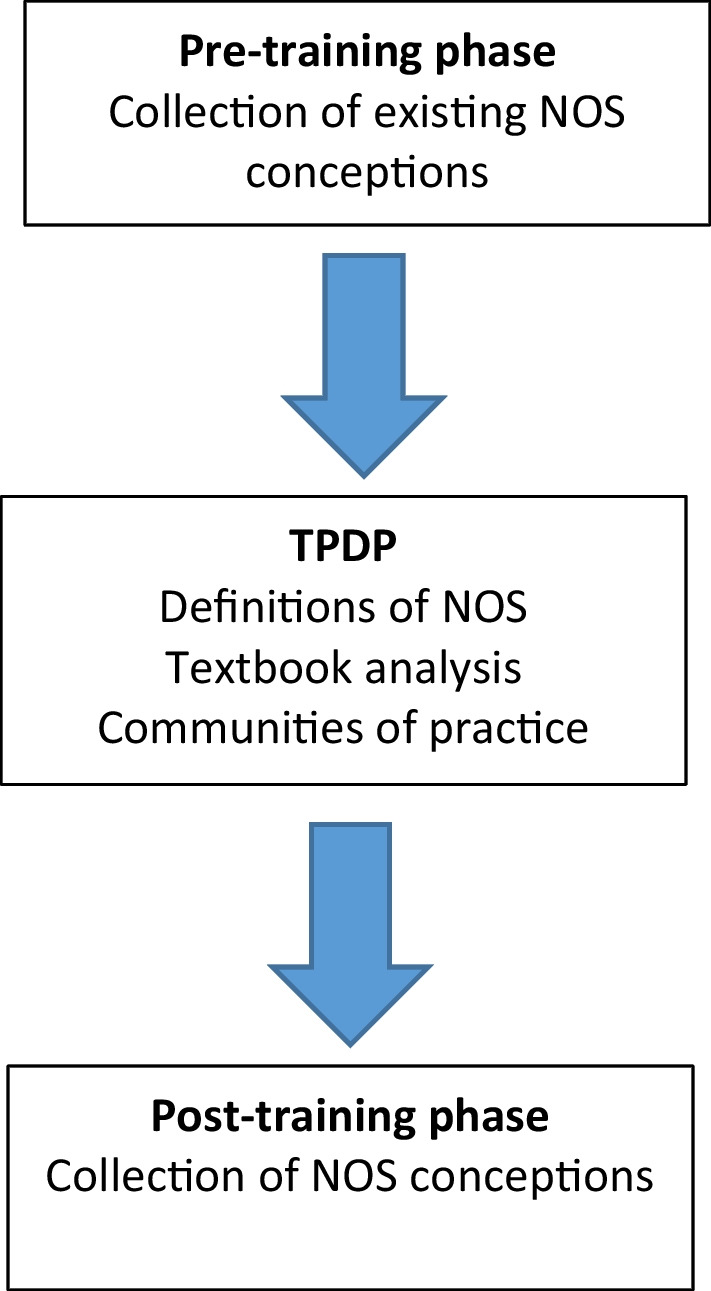
Research process

The outline of the research process is documented in the flow diagram below (Fig. 1).

### Pre-training Phase

To collect the existing conceptions of NOS understanding of the teachers, the IFVNOS questionnaire was distributed via Google docs or email. All teachers completed the forms online and returned them via the same mode. Upon receipt of the completed forms, the researchers analysed the responses and where clarity was required, the researchers developed a unique interview schedule aimed at seeking full understanding of the responses. This data together with the IFVNOS responses were analysed using content analysis. Content analysis is a coding technique by Krippendorff (1980) in which text is compressed into categories stipulated by rules of coding. The text collected from the IFVNOS responses was categorised according to the 11 IFVNOS aspects. These aspects were then analysed and scored according to Abd-El-Khalick’s scoring rubric for NOS. During scoring, a rating from − 3 to + 3 which represents the implicitness or explicitness of the NOS treatment is allocated. The scoring rubric developed by Abd-El-Khalick (2013a, 2013b, 2013c, 2012) is shown below:

- Three points = Explicit, informed, and consistent representation of the target NOS aspect.
- Two points = Explicit, partially informed representation of the target NOS aspect.
- One point = Implicit, informed, and consistent representation of the target NOS aspect.
- Zero points = The target NOS aspect is not addressed.
- Negative one point = Implicit misrepresentation of the target NOS aspect.
- Negative two points = The textbook materials convey mixed explicit and/or implicit messages about the target NOS aspect.
- Negative three points = Explicit, naïve representation of the target NOS aspect (Source: Abd-El-Khalick: NOS textbook analysis methods/ UIUC: April 20^th^, 2013/ Scoring rubric).

In illustrating the scoring rubric, Table 5 shows exemplar IFVNOS responses and corresponding scores that were allocated.

**Table 5:** Exemplar IFVNOS responses and corresponding score allocated

IFVNOS aspect (code)	Responses	Understanding
Empirical	• The study of properties of nature both physical and natural through observations and experiments • It relies on evidence to build its knowledge base • Theories can be enhanced or modified as more information arises due to improved technology	+ 3
Social dimension of science	They may work together because you build knowledge based on what someone else has done, and normally exist in a vacuum. So when someone is working on something, they use other people, so they built up knowledge. And yeah, so you work with others	+ 2
Inferential	• Considering the size of the atom, their theory is based on experiment. They observed particles and their behaviour • You experiment or you test then you draw a conclusion	+ 1
Inferential	• The way they interpret their findings may differ	0
Social and cultural embeddedness of science	• Science should be universal. Newton’s laws are applicable to any culture or political ideology. We dealing with the physical world. We not dealing with context. Water runs downhill and that happens whether we in Africa or whether we in China. Whether Chinese want water to run down is different	− 1
Scientific methods	• No scientific method is not rigid you can start from anywhere e.g. results, observations • They need to observe, astronomy they have to observe • There is no experiment that does not require a hypothesis because what will you be trying to prove. It looks like that experiment will be baseless	− 2
Scientific methods	• All sciences follow the same scientific method they form a hypothesis and then process to test it. The hypothesis is a stated relationship between two variables that can be measured • We will continue to apply the principles of science until we are able to identify this definite concept. That’s why I state in principle we would like to say whatever we have in the field of science conforms to this idea of the scientific method	− 3

### Teacher Professional Development Program on Textbook Analysis

An explicit reflective approach was followed to train teachers on NOS. An explicit reflective approach is one in which the outcome of the training is aimed at understanding NOS aspects which are defined explicitly rather than assuming that NOS understanding is achieved through activities such as scientific inquiry which have other outcomes (Pleasants, 2017). The TPDP was carried out online in two segments. The online mode of interaction was adopted due to the COVID-19-induced lockdown. Segment 1 comprised online training on explicitly defining NOS, identifying NOS aspects in popular media, and conducting textbook analysis on chapters previously selected by the researcher from high school Natural Sciences, Life Sciences and Physical Sciences textbooks for representation of NOS. The online segment was held on Google Meet. In the second segment of the program, subject-specific communities of practice were setup via WhatsApp by the researcher. In these groups, teachers selected text and conducted content analysis of the text for a representation of NOS. This was conducted over a 6-week period.

The TPDP is elaborated below.

#### Segment 1

- a) Defining NOS

**Fig. 2:**
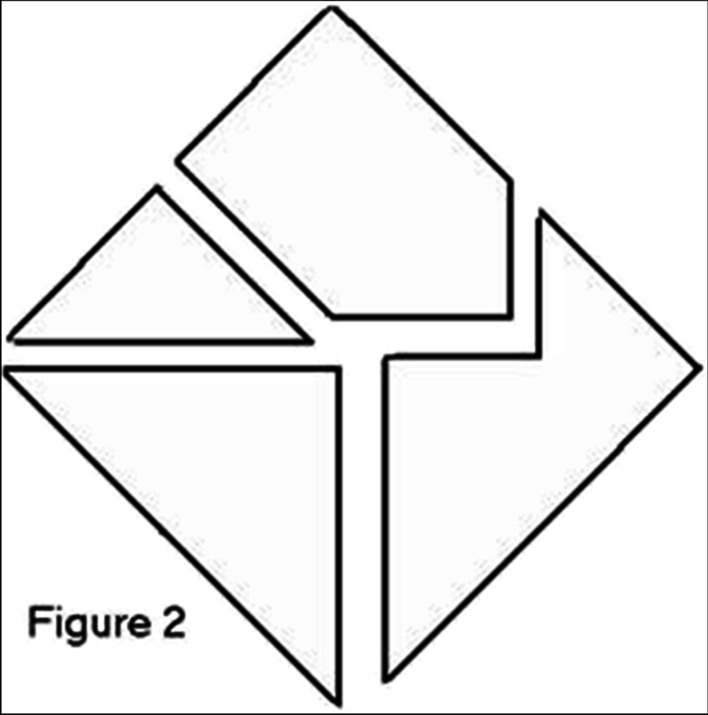
Square puzzle

In introducing NOS, teachers were asked to participate in making a puzzle via an online application puzzle.com. They were provided with puzzle pieces to make the square below (Fig. 2).

**Fig. 3:**
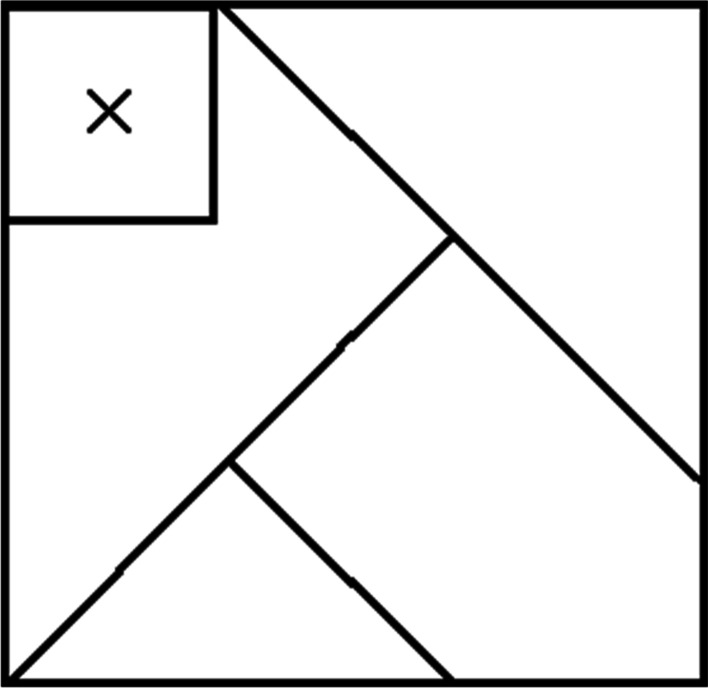
Square puzzle with new piece

A new piece was then introduced to the existing pieces and teachers had to once again piece the puzzle (Fig. 3). See below.

Teachers were asked to comment on how doing this activity can be likened to science. Based on the responses provided by the teachers, the researcher expanded on the responses to provide explicit definitions of NOS. The 11 IFVNOS aspects are presented in Table 2 and the meaning of each aspect was explained to the teachers.

- b) Identifying NOS in a Socio-scientific Issue

**Fig. 4:**
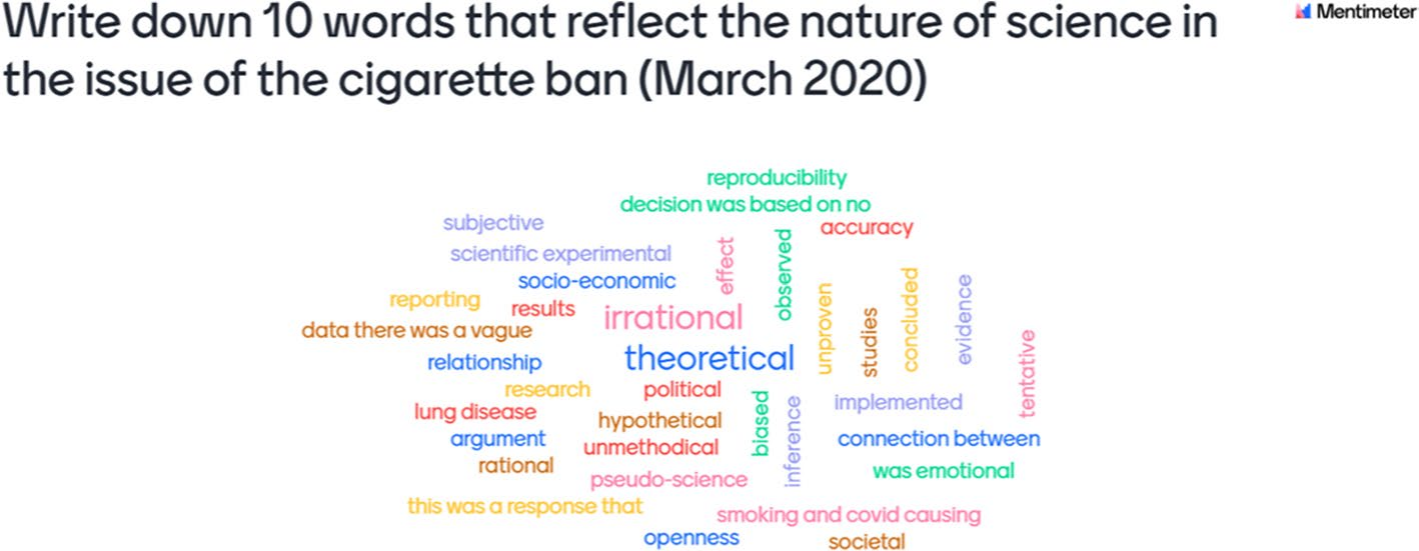
Reflection of NOS in the issue of the cigarette ban

The researcher presented to the teachers a discussion on a socio-scientific issue (SSI). A SSI involves an interaction between science and society and thereby providing a meaningful context for learning (Chowdhury et al., 2020). The topic used was on the “Cigarette ban in South Africa during the 2020 COVID-19 lockdown” (Fig. 4). To facilitate the discussion, the researcher shared material in the form of newspaper articles and videos that represented the views of various stakeholders who were affected by the cigarette ban. The opinions of smokers, small-scale and large-scale tobacco farmers, lawyers and health specialists were presented by the researcher as found on the Internet.

The teachers were asked to identify NOS aspects in the socio-scientific topic presented by the researcher. They took turns to orally share their opinions during the online session.

- c) Textbook Analysis

The researcher selected a history of science article on the history of the microscope from a grade 9 Natural Sciences textbook. Using this article, the researcher introduced textbook analysis to the participant teachers. Using a history of science (HOS) article is one of the approaches documented in literature by Lederman & Lederman (2014) that can improve NOS understanding if coupled with other explicit methods. In this study, content analysis of text is the core explicit approach to teaching NOS. The researcher highlighted text, images or figures that resonated with NOS aspects within the history of the microscope article. For instance, the article documents the different scientists who contributed varying aspects to the development of the microscope over time. This reflected the tentative aspect of NOS. Together with the participant teachers, the entire article was analysed and NOS aspects were inferred from the content. The representation of the NOS aspects as explicit or implicit was then documented. Teachers were provided with exemplar text by Abd-El-Khalick (2013a, 2013b, 2013c, 2012) to differentiate between informed and naïve representations of NOS. Consider the two statements below concerning the social and cultural embeddedness of science:

- Science is about the facts and could not be influenced by cultures and society. Atoms are atoms here in the USA and are still atoms in Russia.
- Of course culture influences the ideas in science. It was more than 100 years after Copernicus that his ideas were considered because religious beliefs of the church sort of favoured the geocentric model.

Statement (a) represents a naïve view of the social and cultural embeddedness of science compared to statement (b) which is considered to be a more informed view.

Following the group analysis of the NOS aspects in the HOS article, the teachers were presented with subject-specific text to analyse individually. Life Sciences teachers received a text on the chapter of Environmental Studies from a matric textbook recommended by some of the teachers. Physical Sciences teachers analysed text on the chapter of the Photoelectric Effect from a grade 12 textbook commonly used by some of the participant teachers. On completion of the individual exercises, the teachers took turns to report their findings on NOS aspects represented in the text provided together with their analysis of whether the treatment is explicit or implicit.

#### Segment 2

In the second segment of the TPDP, small communities of practice were created by the researcher following the online session on introducing NOS and training of textbook analysis. Communities of practice are formed by people who engage in a process of collective learning in a shared domain of human endeavour (Wenger-Trayner & Wenger-Trayner, 2015). The teachers were separated into two groups according to their subject specialities. Two WhatsApp groups were created, one for Life Sciences and the other for Physical Sciences teachers. Each group comprised of five teachers. The teachers took turns over a 6-week period to post the material that they would be teaching in their respective lessons and analysed the content for a representation of NOS aspects. They were asked to use the following prompts to guide their presentations.

- Identify the NOS aspects you would want to include in the topic that you are teaching.
- How will these NOS aspects be included?
- Why should learners know about these NOS aspects?
- What challenges do you foresee when teaching NOS?

The teachers posted various materials ranging from videos to textbook pages, assessment questions and notes.

### Post-training Phase

After the TPDP, the teachers were asked to complete the same IFVNOS questionnaire they had filled in before. The teachers completed the forms online and once again the researchers analysed the responses to allocate NOS aspects to determine NOS understandings of each teacher.

## Findings

### Pre-training Findings

Table 6 presents a deep analysis of the TPDP by detailing the pedagogical objectives of each activity and the findings in relation to these objectives. The 10 teachers were found to have an overall inadequate understanding of NOS. Eleven IFVNOS aspects identified from the questionnaire responses were allocated scores ranging from − 3 to + 3. Each teacher could therefore obtain a cumulative score ranging from − 33 to + 33. The lowest cumulative score for the teachers was − 6, indicating implicit understanding of NOS, whilst the highest score was + 19.

**Table 6:** Teacher professional development program activity analysis table

Activity	Objective	Findings	Discussion of findings
Online puzzle activity	To realise teacher initial ideas of what science is through playing the puzzle	• Solving problems • Trial and error … you would try again until you got it right • You can do science from that from any way that what comes to my mind • It is through observation that you develop a strategy (Representative teacher responses)	The teachers held prior conceptions of what science is and this assisted in shaping the discussion to introduce NOS. The original ideas presented by the teachers were used as a baseline
Socio-scientific topical issue discussion	To identify NOS aspects of social nature of science, empirical, ethical practices, inferential, and science vs pseudoscience	The following NOS tenets were identified by the teachers: empirical, social and cultural embeddedness, science vs. pseudoscience, tentative, theory-driven, inferential, scientific methods and ethical practices	After receiving the formal definitions of the NOS tenets, the teachers could explicitly identify them in the socio- scientific issue presented
Content analysis of textbook materials	To identify NOS aspects in textbooks and realise how it is infused with teaching	• But they have not really showed the change in people’s perspective of what a cell was. And I’m sure there must be literature that represents that. So it does not, in a sense, show the contested nature of science • So knowledge is socially negotiated. There are many people who are involved. It is not just one scientist here. (Representative teacher responses) The following NOS tenets were identified by the teachers: • Empirical • Theory laden • Creative • Scientific theories • Tentative	The teachers were able to not only identify NOS tenets in the texts they analysed but they further proposed ideas on how the tenets could have been more explicitly presented for clearer understanding

Table 7 shows the scores for each teacher on the NOS aspects.

**Table 7:** Pre-intervention IFVNOS scores per teacher

NOS aspect	T1	T2	T3	T4	T5	T6	T7	T8	T9	T10	NOS total
Empirical	2	2	2	2	1	1	3	3	0	2	20
Inferential	3	1	1	1	0	− 1	3	3	0	1	13
Creative	− 2	− 2	− 2	− 2	2	− 1	− 2	1	0	− 2	− 12
Tentative	3	3	− 2	− 2	2	+ 2	2	3	2	3	19
Theory-driven	3	3	3	0	3	− 1	3	2	2	3	22
Scientific knowledge	− 2	− 2	− 2	− 2	2	− 1	− 1	2	− 2	2	− 4
Social dimension	3	3	3	2	2	2	2	1	1	2	23
Social and cultural	2	− 2	3	− 1	3	3	3	3	1	3	21
Science vs pseudo	2	1	1	1	1	0	1	1	0	2	11
Ethical practices	0	2	− 2	− 2	0	1	− 1	2	0	2	2
Science methods	− 3	− 3	2	− 3	2	− 3	− 2	− 2	− 3	− 2	− 19
Cumulative score	**11**	**6**	**7**	− **6**	**18**	**2**	**11**	**19**	**1**	**16**	

The bold scores indicate cumulative scores calculated by adding the preceding scores in the column

It can be seen from Table 7 that the social dimension of science, social and cultural embeddedness of science, theory-driven and empirical NOS aspects were most understood by the participant teachers before the TPDP. Scientific methods followed by the creative aspects of NOS were the most naively understood by the teachers. For example some naïve understandings of scientific methods are reflected in the following responses:

All sciences follow the same scientific method they form a hypothesis and then process to test it. The hypothesis is a stated relationship between two variables that can be measured.

it depends on what is being tested but the ultimate goal is to gather data that is necessary to prove something.

Similarly, for the creative aspects these naïve understandings were revealed:

you were expecting to see something and you get this totally anamolous data you can investigate it and it still does not make any sense you research as much as you can you can go across, nobody has got that data and you repeat it you get the same data, then at that point imagination is necessary

I think you should interpret data that is there. If there is too much creativity in that sense then to me it does not have scientific validity

### Post-training Findings

The IFVNOS questionnaires were distributed online via Google Forms to the participants upon completion of the 6-week communities of practice segment. Eight out of the 10 original teachers who participated in the first two phases of the research completed the post-training phase. Due to personal reasons, two of the teachers (T4 and T6) opted out of the training. The responses received were analysed in an identical method of content analysis as was performed in the pre-training phase. The text was coded according to the 11 IFVNOS aspects as defined in the conceptual framework and scored on a rubric ranging from − 3 for an explicit misrepresentation of the NOS aspect to + 3 for an explicit representation of the NOS aspect. The table below shows the IFVNOS scores of the 8 remaining teachers (Table 8).

**Table 8:** Post-intervention IFVNOS scores per teacher (T)

IFVNOS	T1	T2	T3	T5	T7	T8	T9	T10
Empirical	2	2	1	3	3	3	1	2
Inferential	2	1	0	1	2	2	2	2
Creative	1	2	2	1	2	3	2	1
Tentative	3	3	3	2	3	3	3	3
Theory-driven	2	3	3	3	3	1	2	2
Knowledge	1	1	1	3	1	3	1	1
Social dimension	1	2	2	3	2	3	2	1
Social and cultural	2	1	1	3	2	1	2	− 2
Science vs pseudoscience	1	3	3	1	1	3	1	2
Ethical practices	1	1	1	1	1	2	1	3
Methods	3	3	2	− 2	− 2	2	− 2	− 2
Total	** + 19**	** + 22**	** + 19**	** + 19**	** + 18**	** + 26**	** + 15**	** + 13**

The bold scores indicate cumulative scores calculated by adding the preceding scores in the column

The creative, scientific knowledge, science methods and ethical practices NOS aspects had the greatest improvement in understanding by the teachers, whilst inferential NOS showed no change in understanding. The aspects related to theory-driven, social dimension and social and cultural embeddedness showed a decrease in NOS understanding. For example, an improvement in the creative aspect is reflected in the responses below:

Scientists use creativity before during and after, actually so you can think about how you can conduct an experiment and during an experiment, depending on how it’s it’s going, you can think of a way to make it better even after you find result. You check what went wrong. What can be improved and all that you can use your imagination.

Scientists have to use creativity and imagination because sometimes the evidence they find does not contain all the puzzle pieces needed to create the full picture. Being creative helps to create possibilities of what something looks like or how it works.

**Fig. 5:**
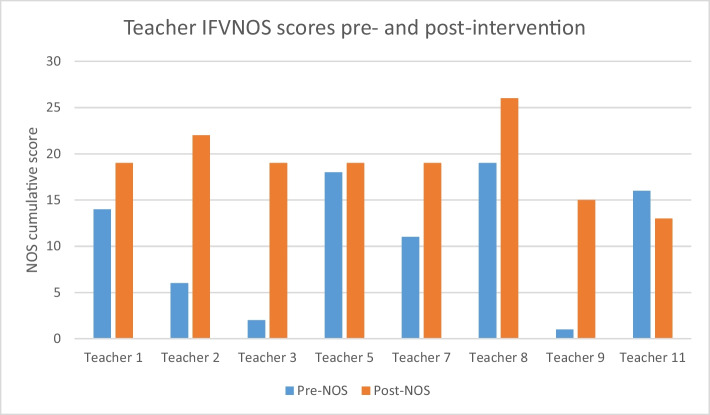
Comparison of pre- and post-intervention intervention IFVNOS scores

All teachers obtained a positive cumulative score for NOS understanding post-training. The higher the score, the greater the NOS understanding. All teachers except for one teacher, teacher 10, increased in their cumulative score for NOS understanding. Teacher 10 showed a decrease from + 16 to + 13. The graph below compares the pre-test scores for NOS understanding with the post test scores (Fig. 5).

## Discussion of Findings

The existing NOS understandings of the teachers before attending the TPDP was found to be consistent with previous researcher findings in South Africa. Teachers generally have a low understanding of NOS (Govender & Zulu, 2017). Post-training findings showed a general increase in NOS understanding in 7 out of the 8 participant teachers. Mesci (2020) shows that not all methodologies will improve NOS understanding. Commensurate with this finding, Abd-El-Khalick & Akerson (2004), Cofre et al. (2019) and Lederman (2007) in studies on improving NOS understanding found that tentativeness, theory/law, subjectivity and socio-cultural embeddedness show little or no improvement in TPDP. Understanding of scientific methods consistently revealed a mixed explicit and/or implicit understanding presented by four teachers in the post-intervention with a score of − 2. Abd-El-Khalick (2013a, 2013b, 2013c, 2012) explains the difficulty in conceptualising a variety of scientific methods as opposed to one scientific method. This is of particular difficulty when this misconception of the existence of this singular scientific method has been engrained in teachers through popular media in science education.

Textbook analysis as an explicit and reflective methodology was shown to improve NOS understanding of sciences teachers. In this methodology, the teachers in this study engaged in a variety of activities ranging from discussions of socio-scientific issues, hands-on puzzle activities and most importantly, content analysis of text from textbooks for a representation of NOS. A wide range of texts was analysed including HOS articles and chapters from topics which the teachers were teaching in their classrooms. The overall improvement in NOS understanding can be ascribed to these activities that supported an explicit and reflective approach to teacher development.

## Conclusion

There are limited studies in South Africa aimed at improving NOS understanding of teachers through professional development programs; however, this study has shown that aspects of NOS such as creative, scientific knowledge, science methods and ethical practices can be improved. Scientific methods continue to be the most difficult aspect to improve.

The findings of the study alluded to recommendations made by Abell et al. (2001) that to improve teachers’ understanding of NOS, the instruction must be embedded in explicit and reflective methodologies which provide the teachers and learners with varying contexts in which to work with key concepts of NOS. Following a study of a comparison of two approaches to developing in-service teachers’ knowledge and strategies for teaching NOS, Vhurumuku & Chikochi (2017) recommended an explicit development of teacher knowledge of history, philosophy and sociology of science to improve subject matter knowledge of NOS. These activities equipped teachers with definitions of NOS aspects and the ability to identify these aspects in various scenarios relevant to society, resulting in an overall improvement in NOS understanding of seven out of eight teachers.

Professional development programmes conducted by renowned scholars over decades of years aimed at improving teachers’ understandings of NOS and their ability to teach it effectively to their learners recommend time for teachers to collaboratively design lessons, share ideas and provide feedback to one another (Akerson & Hanuscin, 2007). The study incorporated this recommendation through the subject-specific WhatsApp groups in which teachers shared their ideas on how to incorporate NOS aspects into their lessons.

It is recommended that professional development programmes to improve NOS understanding employ an explicit, reflective methodology of textbook analysis as was applied in this study. Waltermann & Forel (2015) found that conducting textbook analysis builds a real bridge between theory and practice, helps teachers adapt to new contexts whilst fostering a reflexive approach and promotes a critical understanding of methodologies. Campanile et al. (2015) also proposed that textbook analysis constructs a bridge between the theory of teaching NOS and practice, and so the assumption made in this study, that if teachers analyse textbooks for a representation of NOS this will inculcate knowledge of how to apply NOS concepts into their teaching practices (pedagogy), was proven correct.

The study was limited to 10 teachers who initially participated in the study, but only eight teachers completed the post-intervention phase. Owing to this small sample size, it is difficult to generalise the findings to the entire teacher population of South Africa. Another limitation to the study was the restrictions imposed on schools due to the COVID-19 pandemic at the time of conducting the research. This meant the TPDP could only be conducted online as opposed to in-person as had been initially planned. Erratic network connections negatively impacted three of the teachers who experienced difficulty in actively engaging in the discussions of NOS representation in content being analysed during the two-hour training.
